# DepStep: An Efficient One-Step rRNA Depletion Workflow for RNA Sequencing in Non-model Organisms

**DOI:** 10.21769/BioProtoc.5764

**Published:** 2026-08-05

**Authors:** Suleman M. Qasim, L. Peter Sarin

**Affiliations:** 1 RNAcious Laboratory, Department of Molecular and Integrative Biosciences, Faculty of Biological and Environmental Sciences, University of Helsinki, Helsinki, Finland; 2 Doctoral Programme in Microbiology and Biotechnology, University of Helsinki, Helsinki, Finland; 3 Helsinki Institute of Life Science (HiLIFE), University of Helsinki, Helsinki, Finland

**Keywords:** Transcriptomics, RNA sequencing, rRNA depletion, Bacteria, *Shewanella glacialimarina* TZS-4_T_

## Abstract

RNA sequencing (RNA-seq) has revolutionized transcriptomics, ribosome footprinting, and polysome profiling, providing a wealth of data. Many RNA-based omics typically remove ribosomal RNA (rRNA) or select for messenger RNA (mRNA) prior to sequencing, thereby enriching reads that map to the translationally active part of the transcriptome. Prokaryotic mRNA lacks the 3′ polyadenylated tail, which excludes the use of poly(A)-based selection methods. While commercial rRNA depletion products exist for prokaryotes, their proprietary nature and potential inefficiency with non-model organisms are factors that may limit broad-scale application. To mitigate this issue, we designed DepStep, a consolidated workflow for one-step rRNA depletion using species-specific biotinylated antisense probes for selective hybridization and removal of the target rRNA molecules. As a proof-of-concept, RNA-seq libraries of the psychrophilic gram-negative bacterium *Shewanella glacialimarina* TZS-4_T_ were prepared using both DepStep and a commercial rRNA depletion kit for gram-negative bacteria, to which DepStep was benchmarked. DepStep compares favorably to the commercial depletion kit; it removes >98.6% of the rRNA content in the sample, resulting in sequencing libraries where the coding DNA sequence (CDS) reads account for >80% of the total read count. Importantly, DepStep’s cost-per-sample is three times lower than the commercial kit, establishing DepStep as a simple yet cost-effective alternative to commercial solutions.

Key features

• DepStep is a comprehensive workflow for one-step rRNA depletion in non-model species.

• Guidelines for design and implementation of specific biotinylated antisense probes.

• Compares favorably in benchmarking to commercial products.

• Alternative for budget-conscious labs with 3× lower cost-per-sample.

## Graphical overview


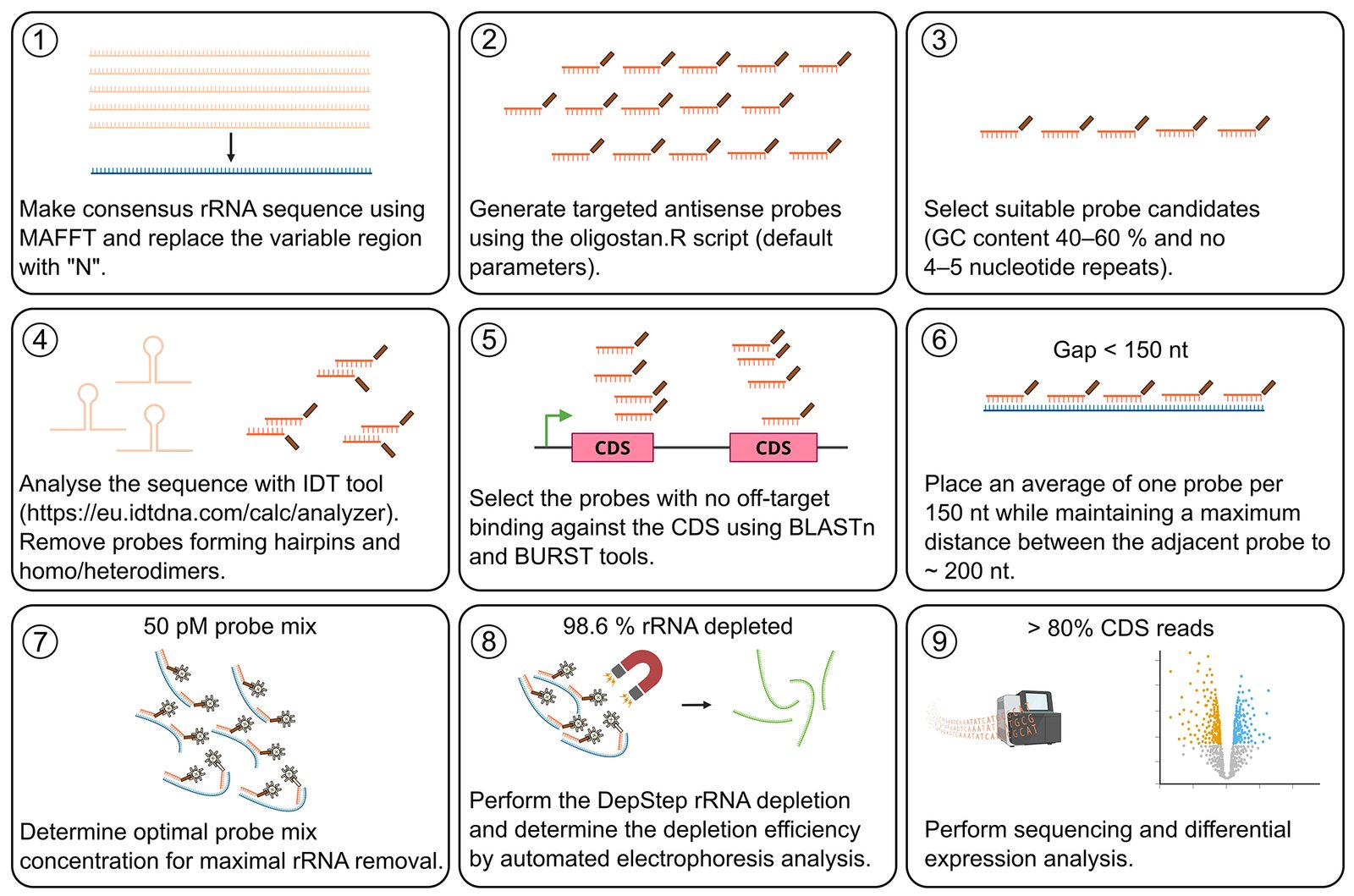


## Background

RNA sequencing (RNA-seq) has become a pivotal tool for generating comprehensive transcriptional profiles and uncovering mechanistic insights into various biological processes [1,2]. However, a significant challenge in RNA-seq is the overwhelming presence of ribosomal RNA (rRNA), which constitutes ~80% of the total RNA in both prokaryotes and eukaryotes [3,4]. This abundance of rRNA can obscure the detection of messenger RNA (mRNA) during sequencing, thereby skewing the transcriptomic data and limiting the insights that can be derived from protein-coding sequences. In eukaryotes, this issue is often circumvented by poly(A) enrichment, a technique that selectively isolates polyadenylated mRNA for sequencing [5]. However, this approach is not applicable to prokaryotes, as most prokaryotic mRNAs lack polyadenylation. Hence, rRNA must be effectively removed from the total RNA to allow accurate quantification of mRNA transcripts.

To achieve this, the preferred option is to rely on commercial rRNA depletion methods, which are available from numerous vendors, particularly when working with established model organisms. On the other hand, commercial kits may be less efficient with non-model organisms, as their depletion strategies might not be as effective in removing rRNA from lesser-studied prokaryotes. Consequently, customized solutions may be needed to remove rRNA, including (i) depletion using rRNA-specific biotinylated probes coupled to streptavidin beads [6–8], (ii) targeted degradation of rRNA–DNA hybrids using RNase H treatment [9], (iii) specific cDNA synthesis from mRNA using non-random primers that do not prime reverse transcription of rRNA molecules [10], (iv) enrichment of mRNA by blocking the amplification of rRNA [11], and depletion of cDNA reads matching rRNA using (v) Taq polymerase endonuclease activity [12] or (vi) Cas9-mediated degradation [13]. While these methods have been successfully used to enhance the capture of mRNA and enable quantitative analysis of mRNA transcripts, they are often laborious to implement, requiring rigorous testing before optimal performance is achieved.

Drawing from these strategies, here we present a simplified one-step rRNA depletion method—DepStep—that utilizes biotinylated antisense rRNA probes linked to streptavidin-coated magnetic beads. Using the non-model bacterium *Shewanella glacialimarina* TZS-4_T_ as proof-of-concept, we designed species-specific rRNA antisense probes and systematically optimized the depletion conditions, removing >98.6% of rRNA molecules without biasing the mRNA and non-coding RNA contents. The subsequent sequencing libraries yielded coding DNA sequence (CDS) reads accounting for >80% of the total read count. To evaluate the effectiveness of our approach, we compared our in-house method to a commercially available rRNA depletion kit that is compatible with our non-model organism. We demonstrate that DepStep offers similar depletion as the commercial kit, with mRNA reads from both methods showing a positive Pearson correlation (r = 0.82). Importantly, we noticed that uniform probe coverage throughout the rRNA molecule is critical for its effective removal, and maintaining a maximum distance of less than 200 nt between adjacent probes resulted in optimal depletion efficiency. Moreover, DepStep can be adapted to generate biotinylated probes for other organisms, facilitating studies on transcriptional regulation across different species. However, its applicability to other organisms may require species-specific optimization. By employing DepStep, we enhanced the detection of mRNA transcripts and increased the overall reads coverage across the CDS region in *S. glacialimarina*. Consequently, DepStep provides a consolidated workflow for generating custom probes, offering an efficient and cost-effective solution for rRNA removal.

## Materials and reagents

**Biological material**

1. *Shewanella glacialimarina* TZS-4_T_ (DSM 115441, HAMBI 3773) [14,15]

**Reagents**

**Buffers, acids, and bases**

1. Tris base [Tris(hydroxymethyl)aminomethane] (Thermo Fisher Scientific, catalog number: BP152-1)

2. Boric acid (Thermo Fisher Scientific, catalog number: B/3800/60)

3. Acidic phenol pH 5.3 (Sigma-Aldrich, catalog number: P4682)

4. MOPS (Thermo Fisher Scientific, catalog number: BP308-100)

5. Ethylenediaminetetraacetic acid (EDTA) (Thermo Fisher Scientific, catalog number: 118432500)

6. Hydrochloric acid (HCl) for pH adjustment (any)

7. Sodium hydroxide (NaOH) for pH adjustment (any)

8. Acetic acid (glacial) (Thermo Fisher Scientific, catalog number: A/0360/PB17)

**Salts**

9. Sodium chloride (NaCl) (Thermo Fisher Scientific, catalog number: S/3120/63)

10. Sodium citrate (Thermo Fisher Scientific, catalog number: BP327-500)

11. Sodium acetate (Thermo Fisher Scientific, catalog number: S/2080/53)

12. Sodium dodecyl sulfate (Thermo Fisher Scientific, catalog number: BP166-500)

13. Guanidine thiocyanate (Thermo Fisher Scientific, catalog number: BP221-1)

14. Ammonium thiocyanate (Thermo Fisher Scientific, catalog number: A/6640/53)

15. Ammonium persulfate (Thermo Fisher Scientific, catalog number: 327081000)

**Organic compounds and solvents**

16. 99.6% EtOH, AA grade (Anora Group Oyj, catalog number: 1025874)

17. 1-bromo-3-chloropropane (BCP) (Acros Organics, catalog number: 106860010)

18. Tween 20 (Thermo Fisher Scientific, catalog number: BP337-500)

19. Formamide (Thermo Fisher Scientific, catalog number: BP228-100)

20. Ficoll 400 (Thermo Fisher Scientific, catalog number: BP525-100)

21. Polyvinylpyrrolidone (Sigma-Aldrich, catalog number: 81420-100G)

22. Formaldehyde (Thermo Fisher Scientific, catalog number: BP531-500)

23. Tetramethylethylenediamine (Thermo Fisher Scientific, catalog number: BP150-20)

24. Glycerol, 99%, analytical reagent grade (Thermo Fisher Scientific, catalog number: G/0650/08)

25. Formaldehyde (Thermo Fisher Scientific, catalog number: BP531-500)

26. Urea (Thermo Fisher Scientific, catalog number: 197460050)

**Media components**

27. Peptone (Sigma-Aldrich, catalog number: 91249-500G)

28. Yeast extract (Thermo Fisher Scientific, catalog number: BP1422-500)

29. Marine broth powder (BD Difco, catalog number: 279110)

30. Agar (Neogen, catalog number: NCM0238A)

**Colorimetric reagents and dyes**

31. Xylene cyanol FF (Thermo Fisher Scientific, catalog number: BP565-10)

32. Bromophenol blue (Thermo Fisher Scientific, catalog number: BP115-25)

**Enzymes**

33. RNasin Plus ribonuclease inhibitor (Promega, catalog number: N2615)

**Probes**

34. Biotinylated rRNA-targeting probes, custom synthesis by Metabion (see Supplementary Table S1 for sequences)

**Gel electrophoresis**

35. Agarose (Thermo Fisher Scientific, catalog number: BP160-100) gel 2%, TAE buffering system for RNA

36. Formaldehyde agarose (Thermo Fisher Scientific, catalog number: BP160-100) gel, 1.5%, MOPS buffering system for RNA

37. Urea polyacrylamide (Thermo Fisher Scientific, catalog number: BP1406-1) gel, 6%, TBE buffering system for RNA

38. Urea polyacrylamide (Thermo Fisher Scientific, catalog number: BP1406-1) gel, 15%, TBE buffering system for RNA

39. Invitrogen SYBR Gold Nucleic Acid Gel Stain (10,000× concentrate in DMSO) (Thermo Fisher Scientific, catalog number: S11494)

40. Midori Green (Nippon Genetics, catalog number: MG04)

41. GeneRuler 1 kb DNA ladder (Thermo Fisher Scientific, catalog number: SM0311)

**Protein solution**

42. Bovine serum albumin (Thermo Fisher Scientific, catalog number: 268130100)

**Blocking of nylon membrane**

43. UltraPure Salmon Sperm DNA solution (Invitrogen, catalog number: 15632-011)

**Solutions**

1. 25% rich marine broth (rMB) media (see Recipes)

2. 2 M NaOH solution (see Recipes)

3. 1 M Tris-HCl pH 7.5 and 8.0 (100 mL) (see Recipes)

4. 0.5 M EDTA pH 7.5 and 8.0 (100 mL) (see Recipes)

5. 5 M NaCl (see Recipes)

6. 5× Tris-borate EDTA (TBE) buffer (see Recipes)

7. 50× Tris-acetate EDTA (TAE) buffer (see Recipes)

8. 20% Sodium dodecyl sulfate (SDS) (see Recipes)

9. 2× RNA loading dye (see Recipes)

10. 10× MOPS buffer (see Recipes)

11. Trizol (see Recipes)

12. 100× Denhardt’s solution (see Recipes)

13. 20× SSC solution (see Recipes)

14. 10× ribohybridization buffer (see Recipes)

15. 1× MyOne B&W buffer + Tween (see Recipes)

16. Probe resuspension buffer (see Recipes)

17. Dynabeads buffer A (see Recipes)

18. Dynabeads buffer B + Tween (see Recipes)

19. Pre-hybridization buffer (see Recipes)

20. Wash buffer (WB 1) (see Recipes)

21. Wash buffer (WB 2) (see Recipes)

22. Wash buffer (WB 3) (see Recipes)

**Recipes**

**1. 25% rMB and solid media**

Weigh 7.5 g of peptone, 1.5 g of yeast extract, and 9.35 g of marine broth powder into a 2 L glass beaker. Add ~800 mL of ddH_2_O and stir using a magnetic stirrer until all components are fully dissolved. Adjust the final volume to 1 L with ddH_2_O. For preparing solid media, add 15 g of agar to 25% rMB media solution and continue stirring while gently heating until the agar is completely dissolved. Store at room temperature (RT) for up to 3 months.

**2. 2 M NaOH solution**

Weigh 8.0 g of NaOH pellets into a 250 mL beaker. Add ~80 mL of nuclease-free ddH_2_O. Use a magnetic stirrer to fully dissolve the pellets. Adjust the final volume to 100 mL. Store at RT for up to 1 year.

**Caution:** The dissolution of NaOH is highly exothermic. Appropriate personal protective equipment, including gloves and safety goggles, should be worn.

**3. 1 M Tris-HCl pH 7.5 and 8.0 (100 mL)**

Prepare Tris-HCl buffers at pH 7.5 and pH 8.0 separately. Weigh 12.11 g of Tris base into a 250 mL beaker. Add ~80 mL of nuclease-free ddH_2_O. Use a magnetic stirrer to dissolve the Tris base. Adjust the pH to the desired value (pH 7.5 and 8.0) with HCl solution. Set the final volume to 100 mL with nuclease-free ddH_2_O using a volumetric flask and filter through a disposable 0.2 μm 150 mL bottle-top filter into a sterile 100 mL glass bottle. Store at RT for up to 1 year.

**4. 0.5 M EDTA pH 7.5 and 8.0 (100 mL)**

Prepare EDTA solution at pH 7.5 and pH 8.0 separately. Weigh 14.61 g of EDTA into a 250 mL beaker. Add ~80 mL of nuclease-free ddH_2_O. Use a magnetic stirrer to dissolve EDTA. Adjust the pH of the solution to the desired value (pH 7.5 and 8.0). Set the final volume to 100 mL with nuclease-free ddH_2_O using a volumetric flask and filter through a disposable 0.2 μm 150 mL bottle-top filter into a sterile 100 mL glass bottle. Store at RT for up to 1 year.

**5. 5 M NaCl (100 mL)**

Weigh 29.22 g of NaCl into a 250 mL beaker. Add nuclease-free ddH_2_O up to 80 mL. Use a magnetic stirrer to fully dissolve NaCl. Adjust the volume to 100 mL in a volumetric flask and filter through a disposable 0.2 μm 150 mL bottle-top filter into a sterile 100 mL glass bottle. Store at RT indefinitely.

**6. 5× TBE buffer (1 L)**

Weigh 54 g of Tris base and 27.5 g of boric acid into a 1 L beaker. Add nuclease-free ddH_2_O up to 800 mL. Use a magnetic stirrer to dissolve the reagents. Once dissolved, add 20 mL of 0.5 M EDTA pH 8.0. Adjust the volume to 1 L in a volumetric flask and filter through a disposable 0.2 μm 1 L bottle-top filter into a sterile 1 L glass bottle. Dilute the stock solution with nuclease-free ddH_2_O to the required concentration. Store at RT for up to 1 year.

**7. 50× TAE buffer (1 L)**

Weigh 242 g of Tris base into a 1 L beaker. Add nuclease-free ddH_2_O up to 800 mL. Use a magnetic stirrer to dissolve Tris base. Once fully dissolved, add 57.1 mL of glacial acetic acid and 100 mL of 0.5 M EDTA, pH 8.0. Adjust the volume to 1 L in a volumetric flask and filter through a disposable 0.2 μm 1 L bottle-top filter into a sterile 1 L glass bottle. Dilute the stock solution with nuclease-free ddH_2_O to the required concentration. Store at RT for up to 1 year.

**8. 20% SDS (100 mL)**

Weigh 20.0 g of SDS into a 250 mL beaker. Add nuclease-free ddH_2_O up to 80 mL. Use a magnetic stirrer to fully dissolve SDS. Adjust the volume to 100 mL in a volumetric flask.

**Critical:** Stir the mixture at 40 °C until clear.

**9. 2× RNA loading dye**

Weigh 1 mg of xylene cyanol FF and 2.5 mg of bromophenol blue. Add 9 mL of formamide, 1 mL of 0.5× TBE, and 25 μL of 20% SDS. Store at RT for up to 3 months or at -20 °C for up to a year.

**10. 10× MOPS buffer (1 L)**

Weigh 41.86 g of MOPS free acid and 4.1 g of sodium acetate anhydrous into a 1 L beaker. Add ~800 mL of nuclease-free ddH_2_O. Use a magnetic stirrer to fully dissolve the reagents. Once dissolved, add 20 mL of 0.5 M EDTA and adjust the pH to 7 with NaOH solution. Set the final volume to 1 L with nuclease-free ddH_2_O using a volumetric flask and filter through a disposable 0.2 μm 1 L bottle-top filter into a sterile 1 L glass bottle. Dilute the stock solution with nuclease-free ddH_2_O to the required concentration. Store at RT for up to 1 year.

**11. Trizol (100 mL)**

Weigh 9.45 g of guanidine thiocyanate and 3.04 g of ammonium thiocyanate into a 250 mL beaker. Add 6.25 mL of 80% glycerol, 3.33 mL of 3 M sodium acetate (pH 5.0), and 10 mL of nuclease-free ddH_2_O. Once dissolved, add 38 mL of acidic phenol (pH 5.3) [16]. Remember that there are two layers in the phenol bottle; take the phenol from the bottom layer using a 25 mL pipette. Set the final volume to 100 mL with nuclease-free ddH_2_O using a volumetric flask. Store at 4 °C for up to 3 months.

**Critical:** Carefully pipette the acidic phenol without aspirating any buffer from the upper layer.

**12. 20× SSC solution (1 L)**

Weigh 175.3 g of NaCl into a 1 L beaker. Add 88.3 g of sodium citrate to the solution. Add nuclease-free ddH_2_O up to 800 mL and dissolve the reagents using a magnetic stirrer. Adjust the solution to pH 7.0 with HCl solution. Adjust the volume to 1 L in a volumetric flask and filter through a disposable 0.2 μm 1 L bottle-top filter into a sterile 1 L glass bottle. Store at RT for up to 1 year.

**Buffers for depletion reaction**

We recommend that buffers 13–17 used in the rRNA depletion reaction be prepared one day before the experiment. However, buffers may be stored at RT for up to one month.

**13. 10× ribohybridization buffer**

**Table:** 

Reagent	Final concentration	Quantity or volume
20× SSC	~19.98×	~9.99 mL
Tween-20	0.1% (v/v)	10 μL
Final volume		10 mL

*Note: Dilute the stock solution with nuclease-free ddH_2_O to the required concentration in the experimental protocol.*

**14. 1× MyOne B&W buffer + Tween**

**Table:** 

Reagent	Final concentration	Quantity or volume
1 M Tris-HCl (pH 7.5)	5 mM	75 μL
0.5 M EDTA	0.5 mM	15 μL
5 M NaCl	1 M	3 mL
Tween-20	0.01%	1.5 μL
Nuclease-free ddH_2_O		Up to 15 mL
Final volume		15 mL

**15. Probe resuspension buffer**

**Table:** 

Reagent	Final concentration	Quantity or volume
1 M Tris-HCl (pH 8.0)	10 mM	0.1 mL
0.5 M EDTA	0.1 mM	2 μL
Nuclease-free ddH_2_O		Up to 10 mL
Final volume		10 mL

**16. Dynabeads buffer A**

**Table:** 

Reagent	Final concentration	Quantity or volume
2 M NaOH	0.1 M NaOH	750 μL
5 M NaCl	0.05 M NaCl	150 μL
Nuclease-free ddH_2_O		Up to 15 mL
Final volume		15 mL

**17. Dynabeads buffer B + Tween**

**Table:** 

Reagent	Final concentration	Quantity or volume
5 M NaCl	0.1 M NaCl	300 μL
Tween-20	0.01%	1.5 μL
Nuclease-free ddH_2_O		Up to 15 mL
Final volume		15 mL

**Buffers for Northern blot**

**18. 100× Denhardt’s solution (250 mL)**

Weigh 5 g of Ficoll 400, 5 g of polyvinylpyrrolidone, and 5 g of bovine serum albumin into a 500 mL beaker. Add ~200 mL of nuclease-free ddH_2_O. Use a magnetic stirrer to dissolve and bring the final volume up to 250 mL with nuclease-free ddH_2_O. Filter through a disposable 0.2 μm 250 mL bottle-top filter into a sterile 500 mL glass bottle. Aliquot the solution into 50 mL Falcon tubes. Store at -20 °C for up to 2 years.

**19. Pre-hybridization buffer**

**Table:** 

Reagent	Final concentration	Quantity or volume
100× Denhardt’s solution	3×	15 mL
20× SSC	5×	125 mL
20% SDS	0.1%	2.5 mL
Nuclease-free ddH_2_O		Up to 500 mL
Final volume		500 mL

We recommend that the pre-hybridization buffer always be prepared fresh.

**20. WB 1**

**Table:** 

Reagent	Final concentration	Quantity or volume
20× SSC	2×	50 mL
20% SDS	0.1%	2.5 mL
Nuclease-free ddH_2_O		Up to 500 mL
Final volume		500 mL

**21. WB 2**

**Table:** 

Reagent	Final concentration	Quantity or volume
20× SSC	1×	25 mL
20% SDS	0.1%	2.5 mL
Nuclease-free ddH_2_O		Up to 500 mL
Final volume		500 mL

**22. WB 3**

**Table:** 

Reagent	Final concentration	Quantity or volume
20× SSC	0.1×	2.5 mL
Nuclease-free ddH_2_O		Up to 500 mL
Final volume		500 mL

**Caution:** SDS in WB 1 and WB 2 is prone to precipitation during storage at RT. If precipitation occurs in the wash buffers, redissolve it by heating to 40 °C.

**Laboratory supplies**

1. Axygen MaxyClear Snaplock microtubes, 1.5 mL (Thermo Fisher Scientific, Axygen, catalog number: MCT150C) or equivalent

2. Glass beads (0.1 and 0.5 mm), nuclease-free (any)

3. 0.2 mL PCR 8-well strips with single flat caps, nuclease-free (Nippon Genetics, catalog number: FG-088WF)

4. 10 μL pipette tips, nuclease-free (any)

5. 200 μL pipette tips, nuclease-free (any)

6. 1 mL pipette tips, nuclease-free (any)

7. Screw cap centrifuge tube, 15 mL (Sarstedt, catalog number: 62.554.002 or equivalent)

8. Screw cap centrifuge tube, 50 mL (Sarstedt, catalog number: 62.547.255 or equivalent)

9. Petri dishes (any)

10. Glass beakers (any)

11. Erlenmeyer flasks (any)

12. Volumetric flasks (any)

13. Glass bottles (any)

14. Dynabeads MyOne Streptavidin C1 (Invitrogen, catalog number: 65002)

15. Zymo-Spin IC Column (ZYMO-RESEARCH, catalog number: C1004-50)

16. RNA Clean and Concentrator kit (ZYMO-RESEARCH, catalog number: R1015)

17. CORALL RNA-Seq V2 Library Prep kit with UDI 12 nt Set A1 (Lexogen, catalog number: 171.96)

18. Str-HRP conjugate (Thermo Fisher Scientific, catalog number: 21130)

19. Amersham Hybond-N+ nylon membrane (Cytiva, catalog number: RPN203B)

20. Whatman paper

21. Enhanced Chemiluminescence kit solutions (Thermo Fisher Scientific, catalog number: 32209)

22. TapeStation Screentapes for RNA (Agilent, catalog number: 5067-5576)

23. RNA ScreenTape sample buffer (Agilent, catalog number: 5067-5577)

24. RNA ScreenTape ladder (Agilent, catalog number: 5067-5578)

## Equipment

1. Fisherbrand Disposable PES bottle top filters 150 mL (Thermo Fisher Scientific, catalog number: 15953307)

2. Fisherbrand Disposable PES bottle top filters 1 L (Thermo Fisher Scientific, catalog number: 15973307)

3. ChemiDoc MP Imaging System (Bio-Rad, catalog number: 12003154 or equivalent)

4. ThermoMixer C (Eppendorf, catalog number: 5382000015 or equivalent)

5. UV crosslinker (Analytic Jena, model: CL-3000)

6. Trans-Blot SD semi-dry transfer cell (Bio-Rad, catalog number/model: 1703957)

7. Electrophoresis Power Supply (CBS Scientific, model: EPS-600 or equivalent)

8. Centrifuge 5427 R with rotor FA-45-24-11 (Eppendorf, catalog number: 5429000010 or equivalent temperature-controlled centrifuge that fits 1.5/2 mL microfuge tubes)

9. Centrifuge 5810 R with rotor fixed-angle FA-45-6-30 (Eppendorf, catalog number: 5811000015 or equivalent)

10. 4150 TapeStation System (Agilent, catalog number: G2992AA)

11. Eppendorf Research^®^ Plus 0.5–10 μL pipette (Eppendorf, catalog number: EP3123000020 or equivalent)

12. Eppendorf Research^®^ Plus 10–100 μL pipette (Eppendorf, catalog number: EP3123000047 or equivalent)

13. Eppendorf Research^®^ Plus 100–1,000 μL pipette (Eppendorf, catalog number: EP3123000063 or equivalent)

14. 744 pH Meter (Metrohm, model: 744 or equivalent)

15. Dual Adjustable Vertical System for polyacrylamide gel electrophoresis (CBS Scientific, catalog number: DASG-250 or equivalent)

16. Agarose gel electrophoresis equipment (any)

17. NanoDrop 2000c Spectrophotometer (Thermo Fisher Scientific, catalog number: ND-2000C or equivalent)

18. Thermocycler with heated lid (Avantor/VWR, catalog number: 732-3428)

19. QuantStudio 3 Real-time PCR system (Thermo Scientiﬁc, catalog number: A28567)

20. Magnetic stand for 0.2 and 1.5 mL tubes (any)

21. Hybridization oven (5–80 °C above ambient) with rotisserie and bottles (any)

22. Incubator shaker New Brunswick^TM^ Excella E25 Shaker (Eppendorf) or equivalent temperature-controlled shaker

## Software and datasets

Antisense probes were designed using default parameters in the oligostan.R script https://bitbucket.org/muellerflorian/fish_quant [17]. The probe candidates were checked for off-target binding using BLASTn v2.10.1 (https://www.ncbi.nlm.nih.gov/) and BURST v0.99.8 [18]. For BLAST, the reference transcripts database was aligned with the candidate probes using BLASTn with the following settings: parameters threshold = 0.05, match score = 2, mismatch score = −3, word size = 1, gap cost = 5, gap extension cost = 2. Probes with 15 or more sequence matches were considered as potential off-targets and removed from the list. Hairpin and homo/heterodimer formation within the probes was evaluated using the IDT OligoAnalyzer tool (https://eu.idtdna.com/calc/analyzer). Each probe’s Gibbs free energy (ΔG) was tabulated in an Excel datasheet, which served as the basis for selecting probes that met the design criteria.

The sequencing reads (FASTQ format) generated in this study were deposited in the European Nucleotide Archive (ENA) under the project accession number PRJEB92206.

## Procedure

**A. Total RNA extraction from *Shewanella glacialimarina* TZS-4_T_**

1. Grow the cells in 50 mL of growth media with constant shaking at 200 rpm (Recipe 1) (25% rMB for *S. glacialimarina*) until the culture reaches the desired OD_600_.

*Note:* S. glacialimarina *culture was grown at 15 °C until it reached an OD_600_ of 0.8. The procedure can be performed with material harvested at any desired OD_600_. Higher OD_600_ might require some additional optimization in the RNA extraction method, such as increasing the volume of acidic phenol or Trizol.*

2. Harvest the cells by centrifugation at 3,200× *g* for 10 min at 4 °C. Carefully decant the supernatant from the tube.

*Note: Keep the cells on ice after harvesting.*

3. Resuspend the harvested cells in 4 mL of 0.9% NaCl solution.

4. Add 4 mL of acidic phenol pH 5.3, 1 mL of Trizol (Recipe 11), and 800 μL of BCP to the cell suspension [15].

5. Add 3 mL of glass beads (0.5 and 0.1 mm in a 1:2 ratio) and lyse the cells in the suspension by vortexing for 10 min.

6. Centrifuge the lysate at 10,000× *g* for 10 min at RT.

7. Collect the aqueous phase and transfer it to a new tube.

**Critical:** Avoid contact with the interphase (i.e., translucent BCP layer).

8. Subject the aqueous phase to a re-extraction step using 2 mL of acidic phenol and 400 μL of BCP. Repeat this re-extraction step a second time.

**Critical:** Avoid contact with the interphase (i.e., translucent BCP layer) during each re-extraction step.

9. Collect the cleared RNA-containing supernatant.

10. Precipitate the total RNA by adding 2.5× volume of 99.6% EtOH to the supernatant.

**Critical:** Mix the sample tubes well.

11. Incubate the mixture at -20 °C overnight.

**Pause point:** The protocol can be paused here for overnight incubation at -20 °C.

12. Pellet the total RNA by centrifugation at 10,000× *g* for 20 min at 4 °C. Carefully decant the supernatant from the tube.

13. Wash the RNA pellets twice using 80% EtOH. Pellet the total RNA after each wash by centrifugation at 10,000× *g* for 20 min at 4 °C. Carefully decant the supernatant.

14. Air dry the RNA pellets.

*Note: Carefully remove the residual EtOH around the pellet using a pipette. Air dry for 20 min at RT.*

15. Resuspend the RNA in nuclease-free ddH_2_O.

16. Confirm the RNA quality by agarose gel electrophoresis. Prepare 2% w/v agarose TAE gel containing 4 μL of Midori Green per 100 mL of gel.

*Note: Prokaryotic rRNA is commonly present in a 1:1 ratio of 23S to 16S, whereas eukaryotic rRNA generally displays a 2:1 ratio of 28S to 18S. These ratios are indicative of a successful RNA extraction with good sample quality. Moreover, a smear below the rRNA bands typically reflects RNA degradation and poor-quality extraction, which may reduce the rRNA depletion efficiency.*

17. Measure the RNA concentration on a NanoDrop UV spectrophotometer.

18. Prepare 1–2 μg of total RNA sample in a 2× RNA loading buffer (Recipe 9). Denature the sample at 80 °C for 5 min. Load the samples into the 2% TAE agarose gel.

**Figure 1.**
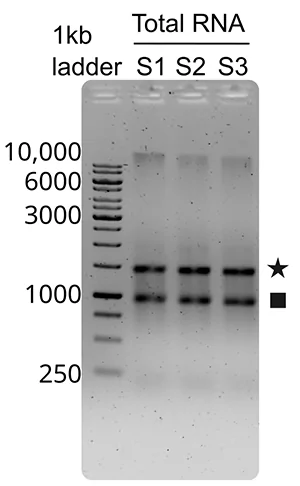
A representative 2% TAE-agarose gel electrophoresis of *S. glacialimarina* TZS-4_T_ total RNA samples (S1–S3). The 1-kb DNA ladder was loaded on the left as a molecular size reference, and RNA bands were visualized using Midori Green dye. The 23S rRNA is indicated by an asterisk and the 16S rRNA by a square.

*Note: Image the gel using a ChemiDoc MP imaging system (or any equivalent device) (Figure 1). Expected outcomes of the total RNA gel electrophoresis are summarized in Table 1.*

**Table 1.** Expected outcome of agarose gel electrophoresis following total RNA extraction

Expected outcome	Result	Conclusion
Prokaryotic 23S and 16S rRNA ratio	1:1	Good quality extraction
Smearing	No	No visible RNA degradation

**B. Probe design for rRNA depletion**

1. Align sequences from the 23S, 16S, and 5S rRNA encoding genes using MAFFT v7.526 to generate a consensus sequence for each rRNA, replacing the variable region with "N" [19].

2. Generate targeted antisense probes from the consensus sequence using the oligostan.R script [17] with default parameters (minimum length = 26, maximum length = 32, ΔG_min_ = -28 kcal/mol, ΔG_max_ = -36 kcal/mol).

3. Screen the generated probes to remove repetitive nucleotide sequences longer than 4–5 nucleotides.

4. Filter the candidates to keep only probes with a GC content between 40% and 60%.

*Note: This yields a total of 83 candidate sequences for* S. glacialimarina: *54 probes targeting 23S rRNA, 27 probes targeting 16S rRNA, and 2 probes targeting 5S rRNA. Please refer to Supplementary Table S1 for complete details on the final selected probes.*

5. Remove probes with a hairpin structure >-3 kcal/mol and a homodimer or heterodimer formation capacity >-10 kcal/mol from the candidate list using the IDT OligoAnalyzer tool (https://eu.idtdna.com/calc/analyzer).

6. Verify that the melting temperature (Tm) of the probes is >70 °C, estimating this based on the Sambrook formula and assuming a 300 mM salt concentration [20].

7. Check the probe candidates for off-target binding using BLAST (https://www.ncbi.nlm.nih.gov/) and BURST v0.99.8 [18,21].

*Note: For BLAST, align the reference transcripts database with the candidate probes using BLASTn with the following settings: parameters threshold = 0.05, match score = 2, mismatch score = -3, word size = 1, gap cost = 5, and gap extension cost = 2.*

8. Remove probes with 15 or more sequence matches from the candidate list, as these are considered potential off-targets (as determined in [22]).

**Figure 2.**
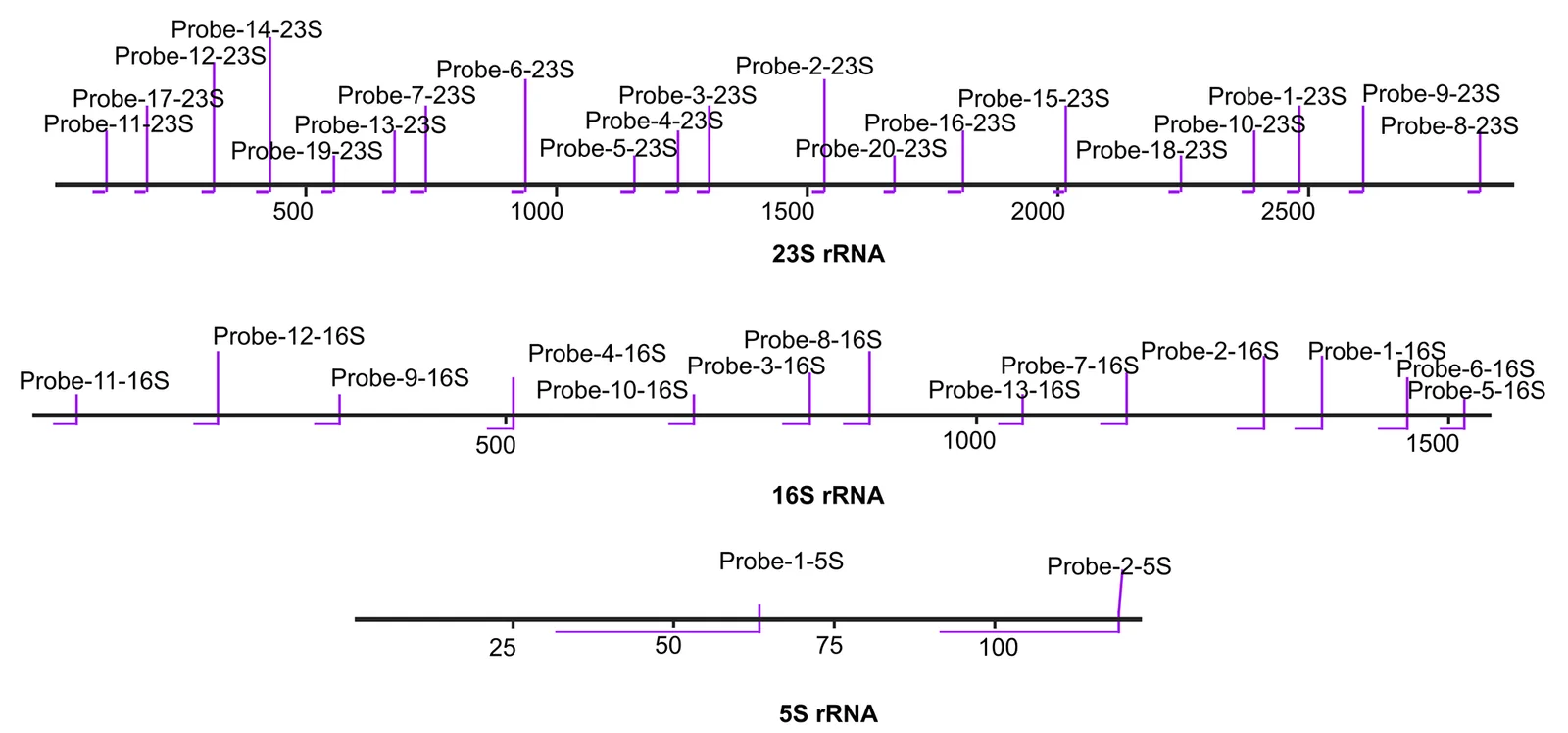
Comprehensive 35-probe set targeting *S. glacialimarina*. There are 20 probes for 23S rRNA, 13 probes for 16S rRNA, and 2 probes for 5S rRNA. Binding sites of the probes are shown using SnapGene version 8.0.1.

*Note: Following the implementation of quality control measures, the final probe library for* S. glacialimarina *contained 35 probes (Supplementary Table 1); 20 targeting 23S rRNA, 13 targeting 16S rRNA, and 2 targeting 5S rRNA (Figure 2). Henceforth, we referred to this method as DepStep-35P.*

9. Maintain an average probe density of one probe per 150 nt rRNA with a maximum distance of 200 nt between adjacent probes.

**Critical:** If the probe design for non-model organisms with divergent rRNA sequences results in limited probe coverage (e.g., gaps >200 nt between adjacent probes), try systematically relaxing the default filtering parameters. For guidance on the prioritization of selection criteria for probe design, please refer to the troubleshooting section (Problem 1).

10. Order the probes from a commercial manufacturer.

*Note: Expected outcomes of the probe design for rRNA depletion are summarized in Table 2.*

**Table 2.** Expected outcome following probe design for rRNA depletion

Expected outcome	Result	Conclusion
Probe candidates	No hairpin, homo/heterodimers	No internal secondary structures or nonspecific binding detected
Coverage	Uniform	Probes are evenly distributed across the rRNA sequence
Off-target binding	None	No probe binding to CDS

**C. Northern blotting**

**C1. Gel and transfer for 23S and 16S rRNA detection**

1. Load 1 μg of total RNA on a 1.5% formaldehyde-agarose gel in 1× MOPS buffer (Recipe 10).

*Note: Prepare the gel and run the sample following standard established RNA laboratory protocols [23].*

2. Transfer the RNA onto a nylon membrane using capillary action with 20× SSC (Recipe 12) as the transfer buffer.

3. Crosslink the RNA to the membrane in a UV crosslinker with RNA side up. Use 120 mJ/cm^2^ energy setting. Cross-link twice.

4. Proceed to step C3.12 for hybridization and detection.

**C2. Gel and transfer for 5S rRNA detection**

1. Load 1 μg of total RNA onto a 15% urea polyacrylamide gel in 0.5× TBE (Recipe 6).

*Note: Prepare the gel and run the sample following standard established RNA laboratory protocols [23].*

2. Visualize the gel on a ChemiDoc MP imaging system or any equivalent device.

3. Presoak the polyacrylamide gel, nylon membrane, and Whatman paper in 0.5× SSC for 5 min.

4. Assemble a transfer sandwich with the Whatman paper on the outer sides and the gel and membrane on the inside.

5. Transfer the RNA onto the nylon membrane using a semi-dry transfer cell for 30 min at 115 mA.

*Note: Calculate and set the total current according to the gel area. We recommend using ~2 mA/cm^2^.*

6. Crosslink the nylon membrane in a UV crosslinker at 120 mJ/cm^2^. Cross-link twice.

7. Proceed to step C3.12 for hybridization and detection.

**C3. Membrane hybridization and detection (for all rRNAs)**

1. Pre-hybridize the membrane using pre-hybridization buffer (Recipe 19) at 45 °C for 2 h.

2. Denature the DNA probe mix (100 pmol) by heating at 70 °C for 3 min. Then, immediately cool on ice.

*Note: The concentration of the probe mix denotes the total concentration of all probes combined, not the concentration of each individual probe. The probes are present at an equimolar concentration.*

3. Denature the salmon sperm DNA by heating it at 98 °C for 5 min and immediately chill it on ice. Perform hybridization by supplementing the pre-hybridization buffer with 200 μg/mL salmon sperm DNA and adding the denatured probes.

4. Incubate the membrane at 45 °C overnight.

**Pause point:** The protocol can be paused here during the overnight incubation at 45 °C.

5. The following day, wash the membrane with a series of stringency washing buffers WB 1, WB 2, and WB 3 for 30 min each at RT (Recipes 20–22).

6. Incubate the membrane in Str-HRP conjugate (1:30,000 dilution) in pre-hybridization buffer (Recipe 19) for 15 min at RT.

7. Mix the Enhanced Chemiluminescence kit (ECL) solutions in a 1:1 ratio and apply them to the membrane.

8. Incubate the membrane for 5 min.

**Critical:** Place the membrane upside down in the ECL solution mix and incubate the membrane away from light. Place the membrane in a ChemiDoc MP imaging system or any equivalent device and set the detection time to 300 s.

**Figure 3.**
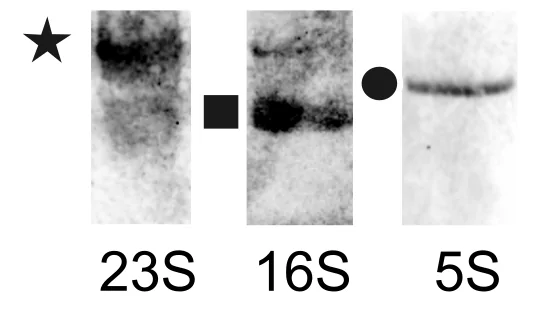
Representative qualitative Northern blot using a 35-probe mix against *S. glacialimarina* rRNAs. The 23S rRNA is indicated by an asterisk, the 16S rRNA by a square, and the 5S rRNA by a circle.

*Note: Qualitative Northern blot analysis confirmed that the DepStep 35-probe mix successfully targeted the 23S, 16S, and 5S rRNA sequences of* S. glacialimarina *(Figure 3). The expected outcome for Northern blotting is summarized in Table 3. For quantitative assessment, image densitometry analysis may be applied to determine probe specificity.*

**Table 3.** Expected outcome for Northern blots

Expected outcome	Result	Conclusion
Probe targeting	Specific	Binds only to the rRNA sequences

**D. rRNA removal from total RNA using DepStep**

1. Mix biotinylated probes targeting rRNA in equimolar amounts to prepare a 100 pmol/μL probe mix solution.

2. Combine 1 μg of *S. glacialimarina* total RNA with the desired amount of the rRNA probe mix (e.g., 5–300 pmol).

*Note: For new organisms, an initial probe concentration can be set at approximately fivefold the molar concentration of rRNA. The upper limit of the probe concentration for optimization is set according to the maximum binding capacity of streptavidin beads used in the depletion step.*

3. Add 2.5 μL of 10× ribohybridization buffer (Recipe 13) [8] and 1 μL of RNase inhibitor. Adjust the final volume to 25 μL with nuclease-free ddH_2_O to set up the hybridization reaction.

*Note: For the control sample, omit the probes from the hybridization reaction, but proceed with all subsequent bead binding and clean-up steps.*

**Critical:** Mix the reaction well by pipetting up and down at least 10 times.

4. Incubate the hybridization reaction for 5 min at 70 °C on a ThermoMixer C without shaking.

5. Mix the reaction with an equal volume (25 μL) of Dynabeads MyOne Streptavidin C1 that have been prewashed according to the manufacturer’s instructions.

6. Thoroughly mix the MyOne Dynabeads stock solution and aliquot 50 μL/reaction to an RNase-free 1.5 mL microfuge tube.

7. Wash the beads three times with 2× original volume (100 μL/reaction) in 1× MyOne B&W buffer + Tween (Recipe 14).

8. Wash the beads two times with 2× original volume (100 μL/reaction) in Dynabeads buffer A (Recipe 16).

9. Wash the beads two times with 2× original volume (100 μL/reaction) in Dynabeads buffer B + Tween (Recipe 17).

10. Wash the beads two times with 2× original volume (100 μL/reaction) in 1× ribohybridization buffer (Recipe 13).

**Critical:** During the bead washing steps (steps D7–10), turn the tubes side to side while they remain inside the magnetic stand to ensure the beads are washed thoroughly within the tube. Repeat this turning motion at least 10 times for each wash step.

11. Resuspend the beads in half of the original volume (25 μL).

*Notes:*

*1. The Dynabeads MyOne Streptavidin C1 beads have a binding capacity of 5 pmol/μL; thus, 50 μL of beads provides a total capacity of 250 pmol for biotinylated probes. The probe concentration should not exceed the bead binding capacity.*

*2. A minimum fivefold molar excess of streptavidin beads over the probe concentration is recommended to ensure efficient capture.*

12. Incubate the samples with the beads on a ThermoMixer C for 15–20 min at 50 °C with constant shaking at 1250 rpm.

13. Place the tubes in a magnetic stand for 5 min to collect the beads.

**Critical:** If the suspension is not completely cleared after 5 min and the streptavidin beads have not fully aggregated on the wall of the tube toward the magnet, leave the tubes in the magnetic stand for 10 min to ensure complete bead collection.

14. Transfer the cleared supernatant into a new tube.

15. Clean up the supernatant using the RNA Clean and Concentrator kit according to the manufacturer’s instructions.

16. Elute the RNA samples from the Zymo-Spin IC column using 10 μL of nuclease-free ddH_2_O.

*Note: The Zymo-Spin IC column generally achieves recovery rates in excess of 90% when a minimum elution volume of 6 μL is applied.*

**Figure 4.**
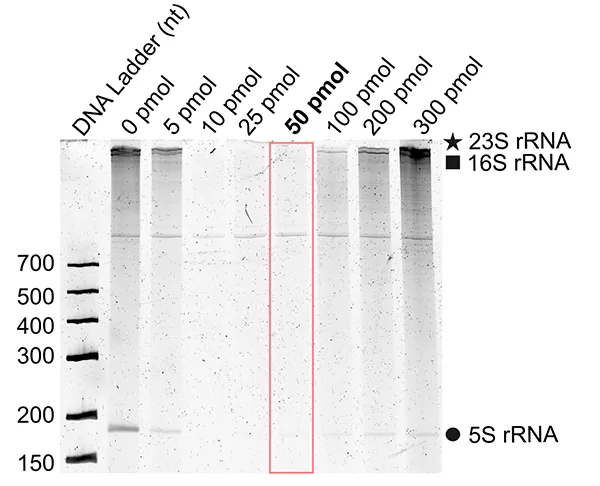
Representative 6% urea polyacrylamide gel electrophoresis of *S. glacialimarina* TZS-4_T_ total RNA samples following rRNA depletion using DepStep-35P with increasing probe amount (from 0 to 300 pmol). The 23S rRNA is indicated by an asterisk, the 16S rRNA by a square, and the 5S rRNA by a circle on the right side. The lane highlighted within the red box corresponds to the 50 pmol DepStep 35 probe mixture selected for RNA sequencing. DNA molecular weight markers are shown on the left side of the gel.

17. Prepare the RNA sample in 2× RNA loading dye (Recipe 9). Load the RNA samples (20 μL) onto a 6% urea polyacrylamide gel.

18. Visualize the gel on a ChemiDoc MP imaging system or any equivalent device. Gel electrophoresis analysis for DepStep 35-probe mix indicated that a probe concentration from 10 to 50 pmol effectively removed the majority of 23S, 16S, and 5S rRNA in *S. glacialimarina* (Figure 4).

*Note: For the final RNA sequencing, 50 pmol of DepStep 35-probe mix per μg of total RNA was selected. The expected outcome for rRNA removal using DepStep is summarized in Table 4.*

**Table 4.** Expected outcome of rRNA removal from total RNA using DepStep

Expected outcome	Result	Conclusion
rRNA removal	No rRNA bands observed in the gel	rRNA was successfully removed

**E. RNA quality control**

*Note: Assess the RNA quality using RNA screen tapes on a TapeStation 4150 system according to the manufacturer’s instructions (summarized below).*

1. Equilibrate the sample buffer at RT for 30 min.

2. Mix 5 μL of the equilibrated sample buffer with 1 μL of the RNA sample in a tube strip.

*Note: Load the RNA screen tape ladder into the first tube of the strip and prepare it identically to the RNA sample (mix 5 μL of sample buffer with 1 μL of the ladder).*

3. Vortex the sample for 1 min.

4. Spin down the tube strip for 1 min.

5. Heat the sample at 72 °C for 3 min using a thermal cycler.

6. Cool the sample at 4 °C for 1 min on the thermal cycler.

7. Spin down the sample again for 1 min.

8. Load the tube screen tape into the TapeStation 4150 system.

9. Following electrophoresis, visualize and analyze the results using the TapeStation Analysis software v.4.1.

**Critical:** The integrity of the total RNA sample should be verified. An RNA integrity number (RIN value) greater than 7 is required.

*Notes:*

**Figure 5.**
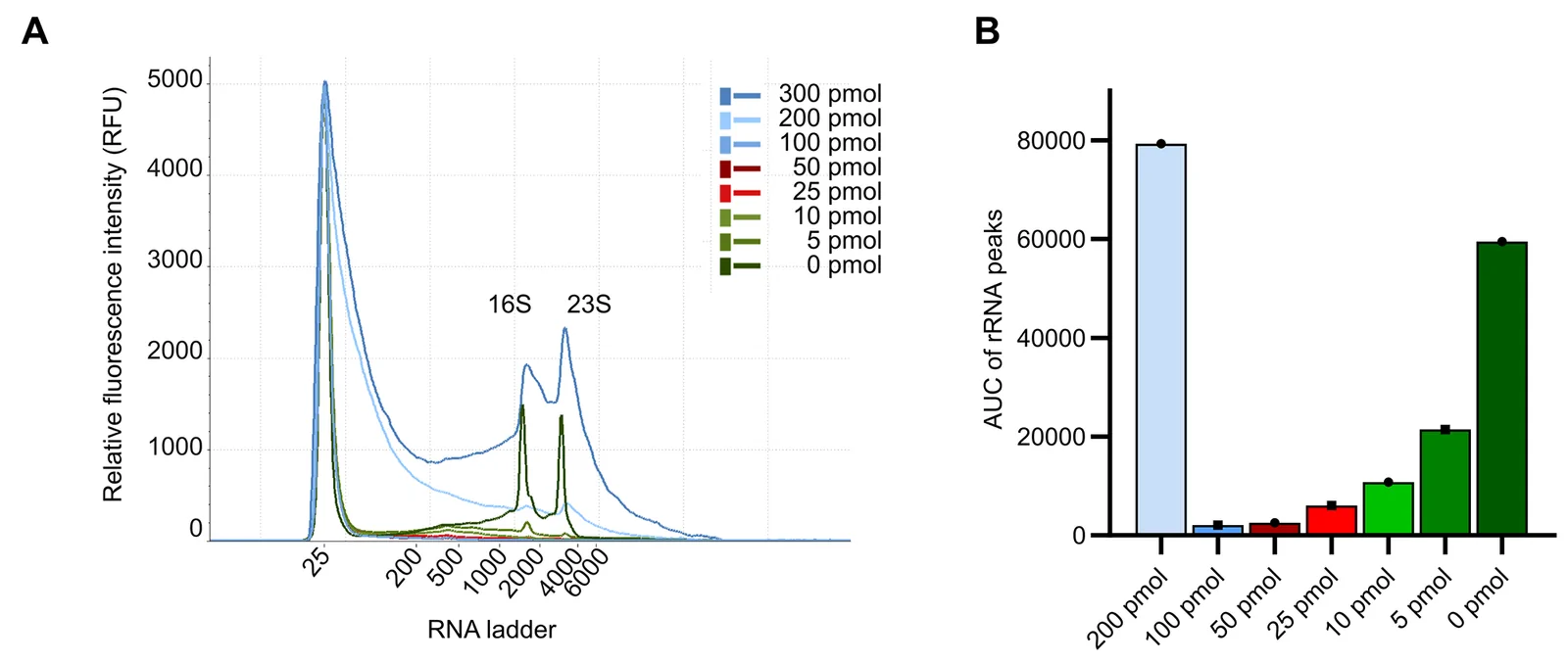
rRNA depletion with DepStep-35P. (A) Electropherogram representation of 23S, 16S, and 5S rRNA depletion upon increasing concentration of DepStep-35P (from 0 to 300 pmol). (B) Bar plot representation of the area under the curve (AUC) of 23S and 16S rRNA from the data-derived TapeStation electropherograms.

*1. The automated electrophoresis electropherogram of the depleted rRNA samples showed a progressive decrease in the amount of 23S and 16S rRNA by up to 98.6% when using 10 pmol, 25 pmol, and 50 pmol probe amounts, respectively (Figure 5A). Area under the curve (AUC) was calculated from 23S and 16S rRNA peaks, showing a stepwise decrease in rRNA (Figure 5B).*

*2. Exceeding the binding capacity of streptavidin beads with an excessive amount of probe (200 and 300 pmol) can lead to bead oversaturation, resulting in inefficient removal of the rRNA–probe complex (Figure 5A, B). See Troubleshooting.*

*3. The expected outcome of RNA quality control following DepStep is summarized in Table 5.*

**Table 5.** Expected outcome for RNA quality control following DepStep

Expected outcome	Result	Conclusion
Validation of rRNA removal	No peaks associated with rRNA	Robust depletion of rRNA

**F. rRNA depletion following RNA quality control**

*Note: rRNA depletion was performed in parallel using DepStep-35P and the commercial rRNA Depletion kit (RiboCop v. 2023) compatible with* S. glacialimarina. *The commercial kit was applied according to the manufacturer’s instructions (summarized below).*

**Figure 6.**
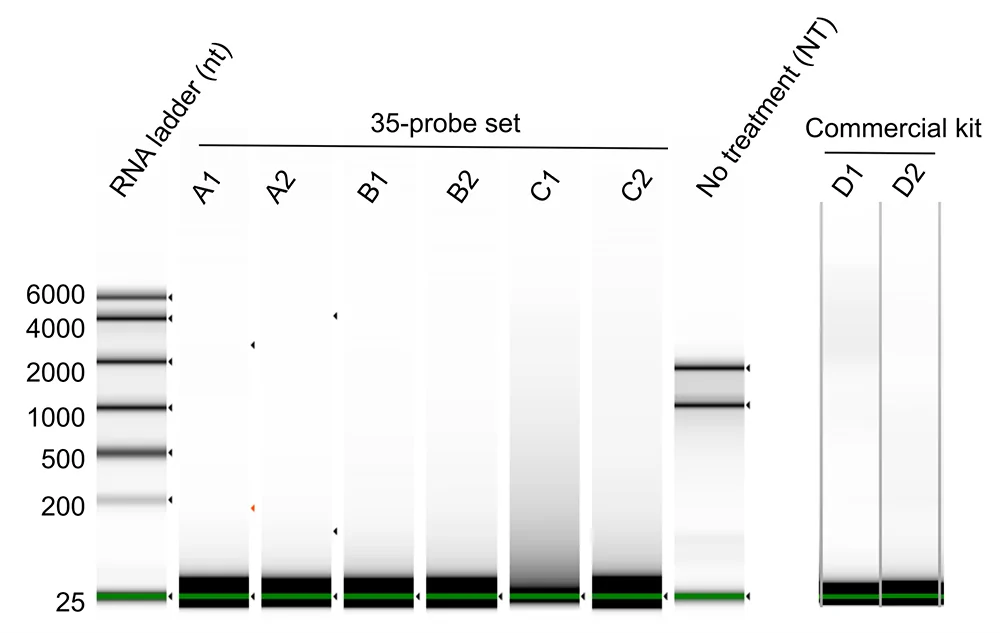
rRNA-depleted samples after DepStep-35P (50 pmol) and the commercial depletion kit. For each sample label, the alphabetical suffix denotes an independent biological sample, whereas the numerical suffix denotes the technical replicate of each biological sample. The green line at the bottom of the gel image corresponds to the low marker band at 25 bp.

1. Dilute 1 μg of *S. glacialimarina* total RNA in 26 μL of nuclease-free ddH_2_O.

2. Add 4 μL of hybridization solution (HS) to the diluted RNA.

3. Add 5 μL of the probe mix for gram-negative bacteria (META, G−) and mix thoroughly.

4. Prepare the rRNA depletion sample and sequencing libraries according to the manufacturer’s instructions.

5. Depletion with 50 pmol of DepStep 35-probe mix per μg of total RNA was assessed by automated electrophoresis analysis alongside the commercial kit (Figure 6).

*Note: If automated electrophoresis indicates incomplete rRNA depletion (e.g., persistent 23S or 16S rRNA peaks), consult the Troubleshooting section for potential causes, including suboptimal probe concentration, insufficient probe density, or bead saturation, and their corresponding corrective actions.*

**G. RNA sequencing**

1. Prepare RNA sequencing libraries from the DepStep samples and total RNA with no treatment (NT) using the RNA-Seq V2 Library Prep Kit with UDI 12 nt Set A1.

*Note: For the total RNA with no treatment (NT) control, use 1 μg of RNA to prepare the sequencing library.*

2. Prepare the sequencing libraries according to the manufacturer’s instructions.

3. Estimate the required amplification cycle number using RT-qPCR with the primers provided in the library preparation kit.

4. Amplify the sequencing libraries using a gradient thermal cycler.

5. Pool the amplified libraries.

6. Sequence the pooled libraries with single-end sequencing for 100 cycles on an Illumina NextSeq2000.

*Note: Following rRNA depletion, libraries can be sequenced using either single-end or paired-end modes with a desired number of cycles on the chosen sequencing platform.*

## Data analysis

1. Demultiplex the raw sequencing reads and convert them to FASTQ format using the bcl2fastq (VBCF Next Generation Sequencing Facility) software.

2. Extract the unique molecular identifiers (UMIs) from the reads using the UMI-tools (v.1.1.5) command umi_tools extract [24].

Script: umi_tools extract -p <umi_pattern> --extract-method=string -I <input_raw.fastq.gz> -S <output_extracted.fastq.gz>

3. Remove adapters using trimmomatic 3.39 with the TruSeq3-SE.fa file [25]. Set the adapter clipping parameters to 2:30:10 (seed mismatches: palindrome clip: simple clip threshold).

Script: trimmomatic SE -phred33 <input_extracted.fastq.gz> <output_trimmed.fastq.gz> TRAILING:<quality_threshold> MINLEN:<length_threshold> ILLUMINACLIP:<adapter_fasta>:2:30:10 2> <trimming_log.txt>

4. Align the trimmed reads back to the *S. glacialimarina* reference genome using the Burrows–Wheeler aligner (BWA-mem v0.7.18) [26].

Script: bwa mem -t <threads> -R "@RG\tID:<sample_id>\tSM:<sample_id>" <reference.fasta> <reads.fastq.gz>

5. Sort the aligned reads according to their coordinates using samtools (v.1.19.2) [27].

Script: samtools sort -@ <threads> -o <output.sorted.bam>

6. Index the bam files using samtools (v.1.19.2) [27].

Script: samtools index -@ <threads> <input_sorted.bam> [output_index.bai]

7. Execute the deduplication script using the UMI-tools (v.1.1.5) command umi_tools dedup [24].

Script: umi_tools dedup -I <input_aligned.bam> -S <output_deduplicated.bam> --temp-dir=<path_to_tmp_directory>

8. Count the reads aligning to each rRNA using samtools (v.1.19.2) [27].

Script 1: samtools view -c -F 4 <input_aligned.bam> # Quantify total mapped reads

Script 2: samtools view -c -F 4 -L <subunit_boundaries.bed> <input_aligned.bam> # Quantify mapped reads overlapping specific ribosomal RNA loci

## Validation of protocol

**DepStep rRNA depletion increases CDS reads to >80% of the total read count**

**Figure 7.**
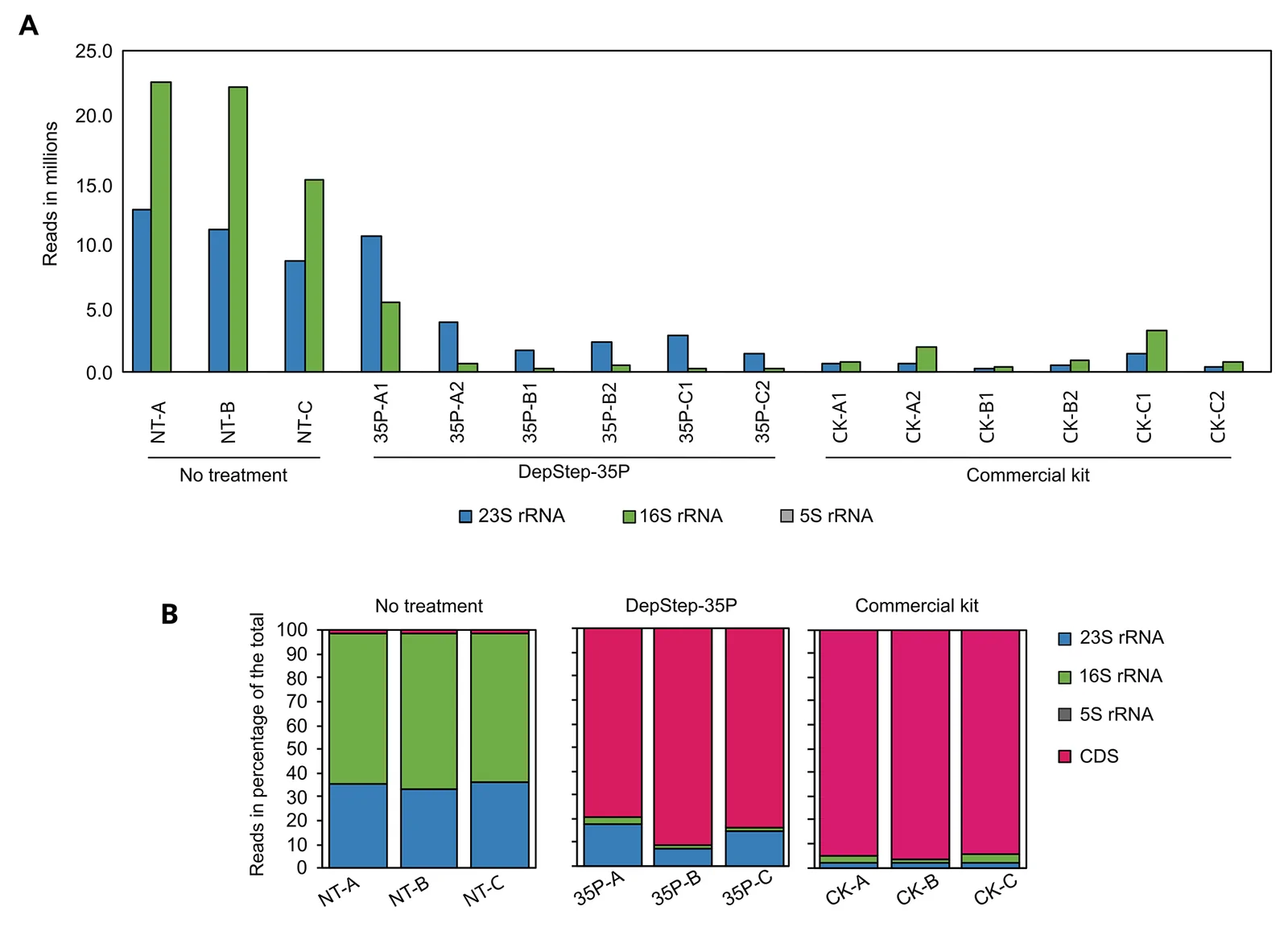
Depletion analysis from DepStep sequencing library preparation of *S. glacialimarina* TZS-4_T_. (A) Raw reads count corresponding to *S. glacialimarina* 23S, 16S, and 5S rRNA in total RNA with no treatment (NT), DepStep-35P (35P) depletion, and commercial kit (CK). For each sample label, the alphabetical suffix denotes an independent biological sample, whereas the numerical suffix denotes the technical replicate of each biological sample. (B) Bar plots depicting raw read counts (in %) mapping to 23S rRNA, 16S rRNA, 5S rRNA, or coding sequences (CDS) in total RNA with no treatment (NT), DepStep-35P (35P), and commercial kit (CK) rRNA-depleted samples. The alphabetical suffixes in the sample labels denote independent biological samples.

Sequencing libraries were prepared using rRNA-depleted samples obtained from DepStep (35-probe mix) and the commercial kit, including libraries from total RNA with no treatment (NT) samples as a control. After sequencing, the reads were mapped back to the reference genome, and the sum of reads aligning to 23S, 16S, 5S rRNA, and the CDS regions was compared between each group of samples. DepStep depletion using the 35-probe mix (DepStep-35P) proved efficient, as reads mapping to rRNA accounted for <20% of the total read count (Figure 7A, B), except for sample 35P-A1, which was identified during TapeStation quality check and excluded (attributed to a technical error) (see Troubleshooting).

**CDS reads obtained from the DepStep-35P and commercial kit show a strong correlation**

**Figure 8.**
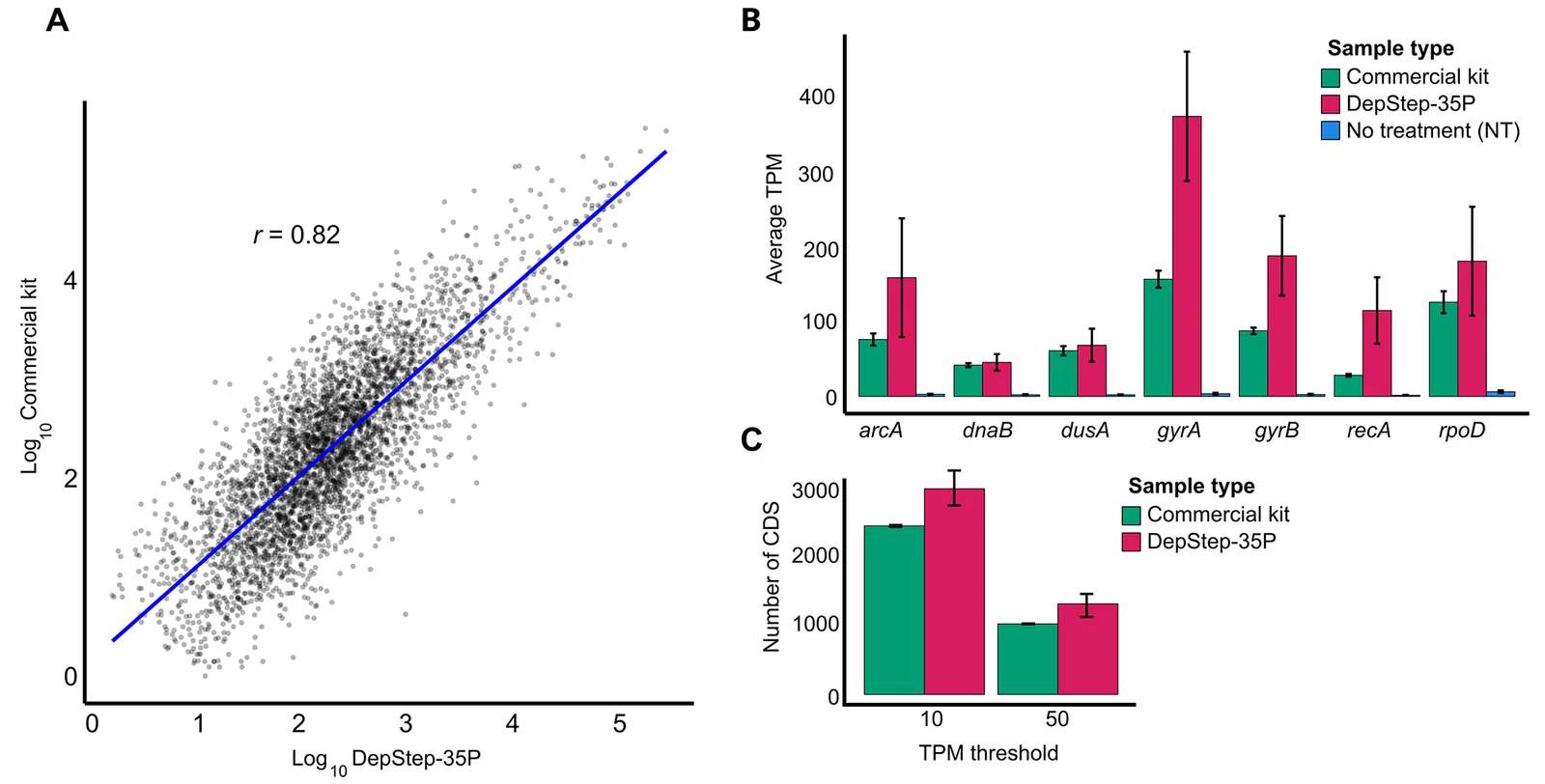
Comparative analysis of coding sequences (CDS) derived from sequencing libraries prepared using DepStep-35P and a commercial kit. (A) Scatterplot showing Pearson’s correlation (r) between DepStep-35P and commercial kit samples. The RNA levels were estimated by averaging normalized log-transformed read counts of three biological replicates for each condition. Lowly expressed genes were removed (log_10_ threshold value < 0.5). (B) Average transcript per million (TPM) of common housekeeping genes in rRNA-depleted samples using DepStep-35P and a commercial kit vs. total RNA. (C) CDS count exceeding the threshold value of 10 and 50 for DepStep-35P and the commercial kit, respectively. Error bars represent the standard deviation of three biological replicates.

Since DepStep-35P and the commercial kit successfully depleted a majority of the rRNA, we expected the relative abundance of mRNA reads to be comparable between both methods, provided that neither approach introduced significant off-target depletion. Indeed, the Pearson correlation (r) value of log_10_-transformed and DESeq2-normalized [28] (method for normalizing count data for differential expression) read counts between DepStep-35P and the commercial kit was 0.82 (Figure 8A). This represents a positive association and suggests that there is no significant difference between these two depletion approaches. Next, we examined the transcript per million (TPM) reads for CDS and focused on CDS associated with known housekeeping genes. Both DepStep-35P and commercial kit samples showed similar TPM trends for each housekeeping gene, albeit the TPM for the DepStep-35P sample was slightly higher than that of the commercial kit (Figure 8B). Upon closer inspection, we noticed that the DepStep-35P sample had a higher number of CDS with TPM values exceeding 10 and 50, indicating that DepStep has a slightly broader dynamic range toward less abundant transcripts (Figure 8C).

**DepStep rRNA depletion has a favorable per-sample cost compared to commercial alternatives**

The primary cost in DepStep is attributed to the use of species-specific biotinylated probes and streptavidin beads. Combined, the total material cost is ~20/reaction, i.e., significantly less than 62/reaction for the commercial alternative used in this study. Although DepStep has a favorable cost-to-performance ratio, it is worth noting that it may be less labor-intensive to opt for a commercial kit, provided the product is compatible for use with the organism-of-study. All materials were purchased from local vendors, and reported costs were calculated at time of experimentation.

## General notes and troubleshooting

**General notes**

1. Clean the work surface with 3% H_2_O_2_ before working with RNA.

2. Always use RNase-free centrifuge tubes while working with RNA.

3. Keep the pipettes clean and RNase-free.

4. Always store the RNA at -70 °C and thaw on ice.

5. Avoid excessive freeze-thawing of RNA samples.

6. Mix the samples thoroughly during rRNA bead hybridization with the antisense probe.

**Troubleshooting**

**Problem 1:** Probe design for non-model organisms results in either inadequate target coverage, increased off-target hybridization, low melting temperatures, formation of unfavorable secondary structures, or probe intrinsic dimerization.

Possible cause: In organisms with divergent rRNA sequences or atypical nucleotide compositions (e.g., extreme AT or GC content), rigid filtering parameters may exclude too many candidate probes, leading to insufficient coverage and large untargeted regions (>200 nt).

Solution: When adapting DepStep to organisms with divergent rRNA, uniform probe coverage must be prioritized to prevent carryover of rRNA fragments. If default parameters limit the coverage, filtering criteria should be relaxed stepwise in line with the recommendations in Table 6.

**Table 6.** Troubleshooting common probe design failures and filtering prioritization

Probe design failure	Corrective action and prioritization guidance
Insufficient probe coverage (gaps > 200 nt)	Highest priority to resolve. Do not proceed with large gaps, as unprobed regions will result in retained rRNA fragments in the sequencing library. To close gaps, systematically relax the criteria below in order of least to most detrimental to the protocol.
Low melting temperatures and extreme GC content	First criterion to relax. For AT-rich organisms, obtaining probes with 40%–60% GC content and Tm > 70 °C may be impossible. Broaden the acceptable GC content range (e.g., 30%–70%) and accept slightly lower Tm probes to bridge coverage gaps. If lowered, you may need to empirically optimize hybridization temperatures during the depletion step to accommodate the lower Tm.
Unfavorable secondary structures	Second criterion to relax. You can marginally relax limits on internal hairpins (ΔG > -3 kcal/mol) to rescue a required probe. However, strictly maintain the homo/heterodimer limits (ΔG > -10 kcal/mol). Strong probe dimers will sequester the probes away from the rRNA target, severely reducing depletion efficiency.
Excessive off-target matches	Strictest criterion (do not relax). Probes with ≥15 sequence matches against the reference transcriptome must be discarded to prevent the unintended depletion of valuable mRNA (CDS) reads. If a critical probe covering a gap exhibits off-target binding, manually adjust its length or shift its target site by a few nucleotides to disrupt the sequence matching the off-target transcript.

**Problem 2:** Transcriptome analysis reveals biases, such as an unexpected reduction in specific mRNA reads (CDS) or skewed transcript per million (TPM) values for non-target genes following rRNA depletion.

Possible cause: Off-target binding of the DepStep antisense probes to non-rRNA sequences within the transcriptome.

**Figure 9.**
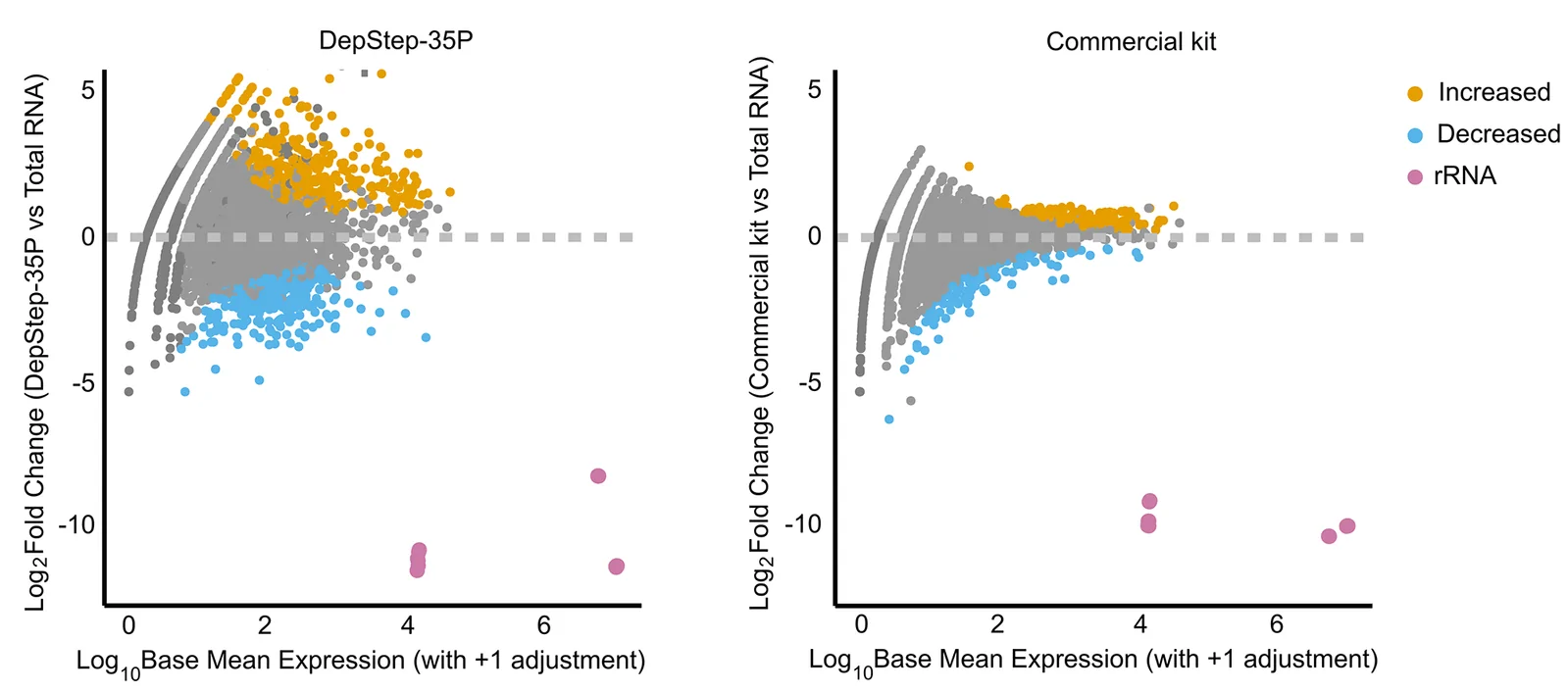
Mean average (MA) plot comparing DepStep-35P (left panel) with the commercial rRNA depletion kit (right panel) with respect to total RNA. The coding sequences (CDS) fold change (vs. total RNA) for DepStep-35P and the commercial kit are represented as yellow (increase) or light blue (decrease) dots, respectively. Magenta dots represent rRNA reads, which are significantly reduced in the rRNA-depleted sequencing libraries.

Solution: Prior to finalizing the probe library, strictly screen the depletion probe candidate sequences against your target organism's transcriptome in silico (e.g., using BLASTn). Ensure you exclude any probes that demonstrate significant homology to CDS regions or exhibit other significant off-target effects. Following sequencing, you can verify specificity by performing a differential expression analysis to confirm that only the targeted rRNA reads are significantly reduced while the TPM of other reads increases or remains stable (Figure 9).

**Problem 3:** Retention of rRNA fragments during depletion, characterized by a high number of sequencing reads aligning to distinct locations within the rRNA in the final libraries.

Possible cause: Low-quality input total RNA sample and lack of uniform probe coverage across the target rRNA sequences. If the distance between adjacent probes is too large, it leaves distinct portions of the rRNA untargeted, leading to retention of smaller rRNA fragments.

Solution: Use RNA with a RIN value above 7 and implement uniform probe distribution across the target rRNA sequences during probe design. The probe set should target approximately one probe per ~150 nt, ensuring that the average inter-probe distance does not exceed 200 nt. To prevent proceeding with suboptimal samples, always perform a sample quality control step using an Agilent TapeStation to identify residual rRNA fragmentation prior to finalizing your sequencing libraries.

**Figure 10.**
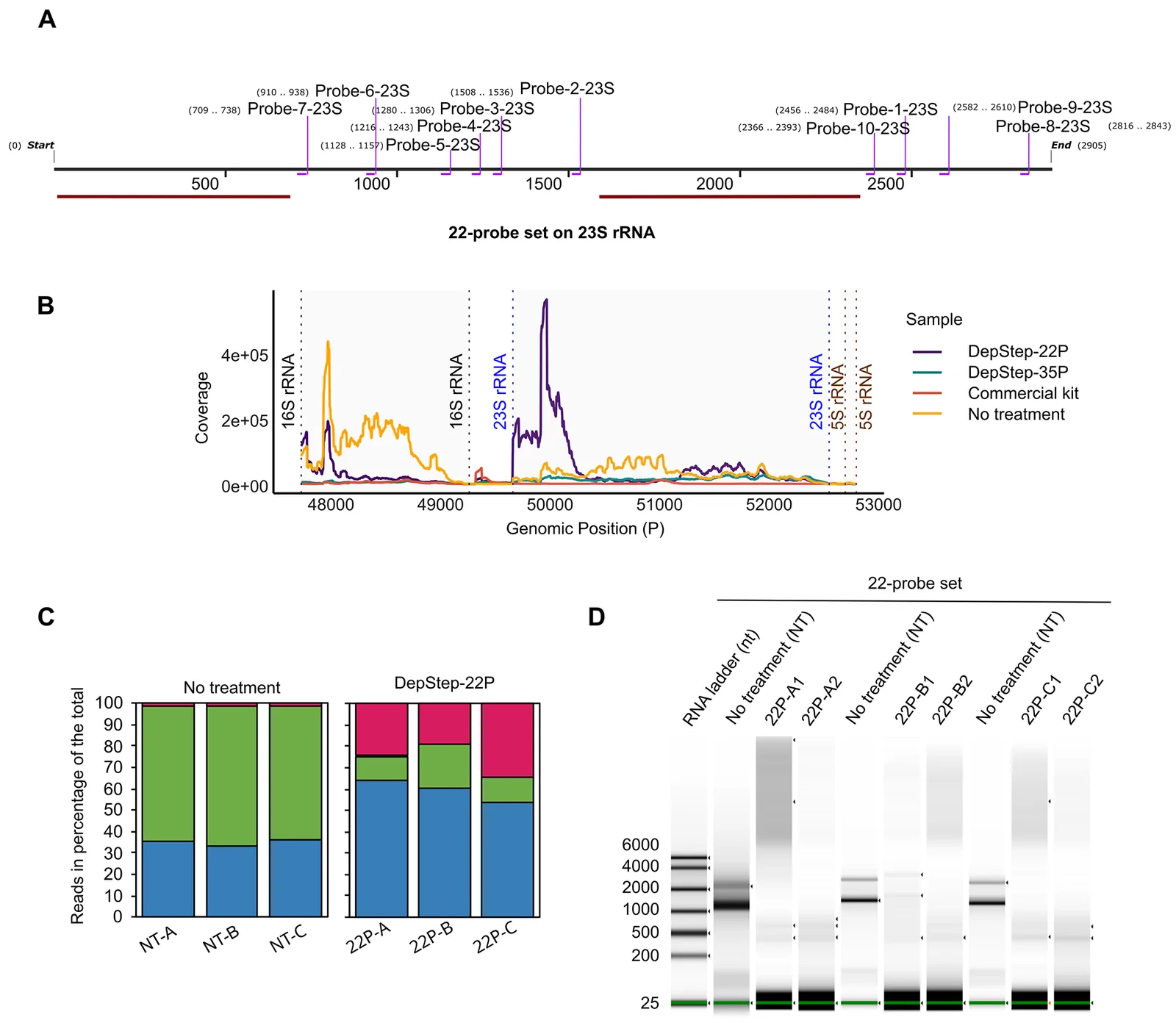
Depletion of rRNA using limited 22-probe set mixes. (A) *S. glacialimarina* 23S rRNA with 22-probe set (10 probes for 23S rRNA) binding sites shown using SnapGene version 8.0.1. The two sites on the 22-probe set not covered by the probes are shown in red. (B) Coverage plot of reads aligning to 23S, 16S, and 5S rRNA sequences in *S. glacialimarina* in DepStep-22P, DepStep-35P, commercial kit, and total RNA with no treatment. (C) Bar plots depicting raw read counts (in %) mapping to 23S rRNA (shown in blue), 16S rRNA (shown in green), 5S rRNA, or coding sequence (CDS) (shown in magenta) in total RNA with no treatment (NT) and DepStep-22P (22P) rRNA-depleted samples. (D) Automated electrophoresis gel analysis of rRNA-depleted samples after depletion with 22-probe set mix. Two bands appear in all samples after the depletion reaction. Total RNA with no treatment (NT) samples are included as controls. The alphabetical suffix in the sample label denotes an independent biological sample, whereas the numerical suffix denotes the technical replicate of the biological sample. The green line at the bottom of the gel image corresponds to the low marker band at 25 bp.

During the development of the DepStep workflow, we assessed a limited 22-probe set featuring the most optimal probe designs (10 probes for 23S rRNA, 10 probes for 16S rRNA, and 2 probes for 5S rRNA). However, the 22-probe set (DepStep-22P) was largely inefficient in removing rRNA, as most reads aligned to two distinct locations within the 23S rRNA (Figure 10A–B). These locations were not covered by DepStep-22P, and the residual rRNA originated from these uncovered regions, leading to a 23S rRNA fragment retained in the final sequencing libraries (Figure 10C). Implementing an Agilent TapeStation-based QC step before sequencing would help detect incomplete rRNA depletion, as shown for DepStep-22P (Figure 10D).

**Figure 11.**
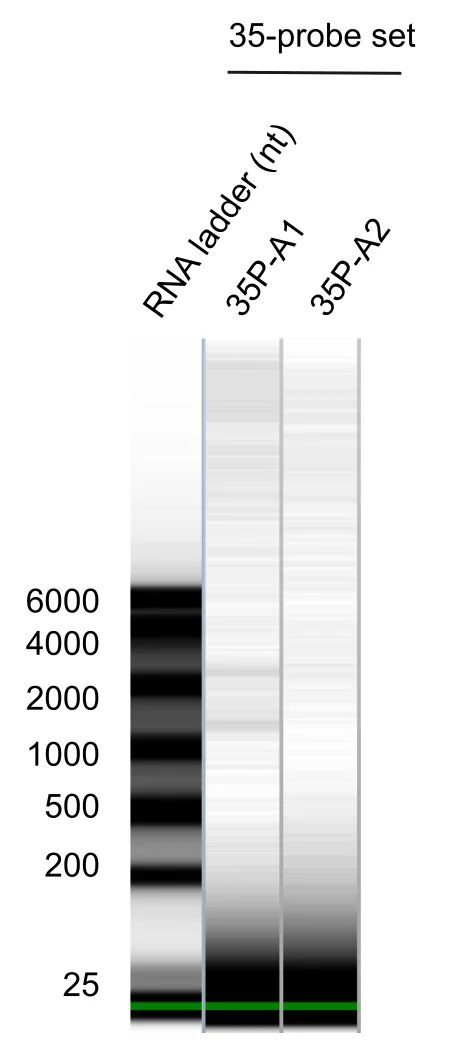
Quality control analysis using DepStep-35P was performed on an electrophoresis gel image with oversaturated contrasts, revealing one sample (35P-A1) with slight depletion inefficiency likely due to technical bias. The alphabetical suffix in the sample label denotes an independent biological sample, whereas the numerical suffix denotes the technical replicate of the biological sample. The green line at the bottom of the gel image corresponds to the low marker band at 25 bp.

**Problem 4:** Incomplete rRNA depletion, indicated by the presence of visible rRNA bands in gel electrophoresis or automated electrophoresis analysis (Figure 11).

Possible cause: Suboptimal probe concentration relative to the input total RNA amount during the depletion reaction.

Solution: Titrate and optimize the probe amount used relative to your input RNA. When depleting 1 μg of total RNA, applying 10–50 pmol of the probe mix effectively removes the majority of rRNA, with 50 pmol achieving up to 98.6% reduction. Furthermore, if you are benchmarking your protocol against commercial products, ensure you test their compatibility with your target organism prior to full-scale use, as different brands and iterations (e.g., QIAseq FastSelect vs. RiboCop v. 2023) can exhibit drastically different levels of success on non-model species.

**Problem 5:** Incomplete rRNA depletion characterized by retained rRNA in the final sequencing library or visible rRNA peaks during quality control, specifically caused by bead saturation during the capture step.

Possible cause: The total concentration of the biotinylated rRNA-targeting probe mix exceeds the maximum binding capacity of the streptavidin-coated magnetic beads. When the beads become saturated, they cannot capture all the hybridized rRNA–probe complexes, leaving targeted rRNA molecules behind in the sample.

Solution: Ensure that the total probe concentration used in the depletion reaction does not exceed the binding capacity of your beads. The Dynabeads MyOne Streptavidin C1 beads used in this protocol have a binding capacity of 5 pmol/μL; therefore, the standard 50 μL bead volume provides a total capacity of 250 pmol for biotinylated probes. To ensure efficient capture of the targeted rRNA, it is highly recommended to maintain a minimum fivefold molar excess of streptavidin beads over the probe concentration. If your optimization requires a higher probe concentration that approaches or exceeds this 250 pmol limit, you must scale up the volume of the streptavidin beads accordingly to prevent saturation.

## Supplementary information

The following supporting information can be downloaded here:

1. Supplementary Table S1. List of DepStep-35P probe sequences used for *S. glacialimarina* rRNA depletion
